# Supplementary material for: A Novel Nanocomposite of Activated Serpentine Mineral Decorated with Magnetic Nanoparticles for Rapid and Effective Adsorption of Hazardous Cationic Dyes: Kinetics and Equilibrium Studies
Source: Nanomaterials (Basel). 2020 Apr 5;10(4):684. doi: 10.3390/nano10040684 (PMC7221753; doi:10.3390/nano10040684)
Supplement: Supplementary file 1 [file nanomaterials-10-00684-s001.pdf]

nanomaterials

Supplementary Materials:

A Novel Nanocomposite of Activated Serpentine Mineral Decorated with Magnetic Nanoparticles for Rapid and Effective Adsorption of Hazardous Cationic Dyes: Kinetics and Equilibrium Studies

Moaaz K. Seliem [1,*], Mariusz Barczak [2], Ioannis Anastopoulos [3] and Dimitrios A. Giannakoudakis [4]

[1] Faculty of Earth Science, Beni-Suef University, 621 Egypt; debakyms@yahoo.com
[2] Department of Theoretical Chemistry, Institute of Chemical Sciences, Faculty of Chemistry, Maria Curie-Sklodowska University, 20-031, Lublin, Poland; mbarczak@umcs.pl
[3] Department of Chemistry, University of Cyprus, P.O. Box 20537, Cy-1678 Nicosia, Cyprus; anastopoulos_ioannis@windowslive.com
[4] Institute of Physical Chemistry, Polish Academy of Sciences, Kasprzaka 44/52, 01-224 Warsaw, Poland; dagchem@gmail.com
* Correspondence: debakyms@yahoo.com

Table S1. Kinetics models for dye adsorption by MNP/SP composite.

Kinetic Model	Formula	Parameters	Refs.
Pseudo- first order	$\ln(q_e - q_t) = \ln q_e - k_1 t$ (Linear)<br>$q_t = q_e(1 - e^{-k_1 t})$ (nonlinear)	q_t (mg g^{-1}): removed amount of basic dye at time t.<br>q_e (mg g^{-1}): equilibrium adsorption uptake<br>k_1 (g mg^{-1} min^{-1}): rate constant of the first-order adsorption.	[25]
Pseudo- second order	$\frac{t}{q_t} = \frac{1}{k_2 \cdot q_e^2} + \frac{t}{q_e}$ (Linear)<br>$q_t = \frac{q_e^2 k_2 t}{q_e k_2 t + 1}$ (nonlinear)	k_2 (g mg^{-1} min^{-1}): rate constant of the second-order adsorption	[26]
Intra-particle diffusion	$q_t = k_p t^{1/2} + C$	k_p (mg g^{-1} min$^{-0.5}$): intra-particle diffusion rate constant.<br>C (mg g^{-1}): The intercept	[27]

The Langmuir [28] and Freundlich [29] equilibrium adsorption models used to fit the adsorption data:

$$q_e = \frac{q_{\max} K_L C_e}{(1 + K_L C_e)} \quad \text{(Langmuir)} \qquad \text{(E1)}$$

$$\text{(Freundlich)} \qquad \text{(E2)} \; q_e = K_F C_e^{1/n}$$

where $q_{\max}$ (mg g^{-1}) is the maximum adsorption capacity and K_L - Langmuir constant, while n and K_F are the constants of Freundlich model.

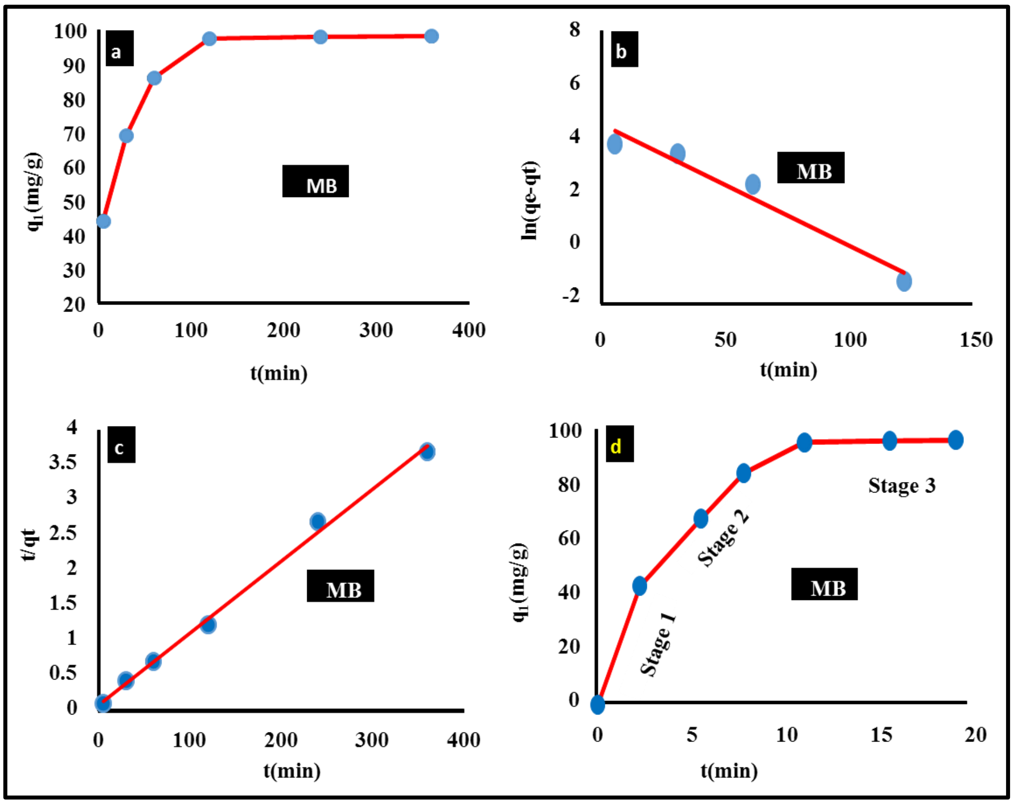

Figure S1. Effect of contact time (**a**), fitting the kinetic data with pseudo-first-order (**b**), pseudo-second-order (**c**), and intra-particle diffusion model (**d**) on MB uptake.

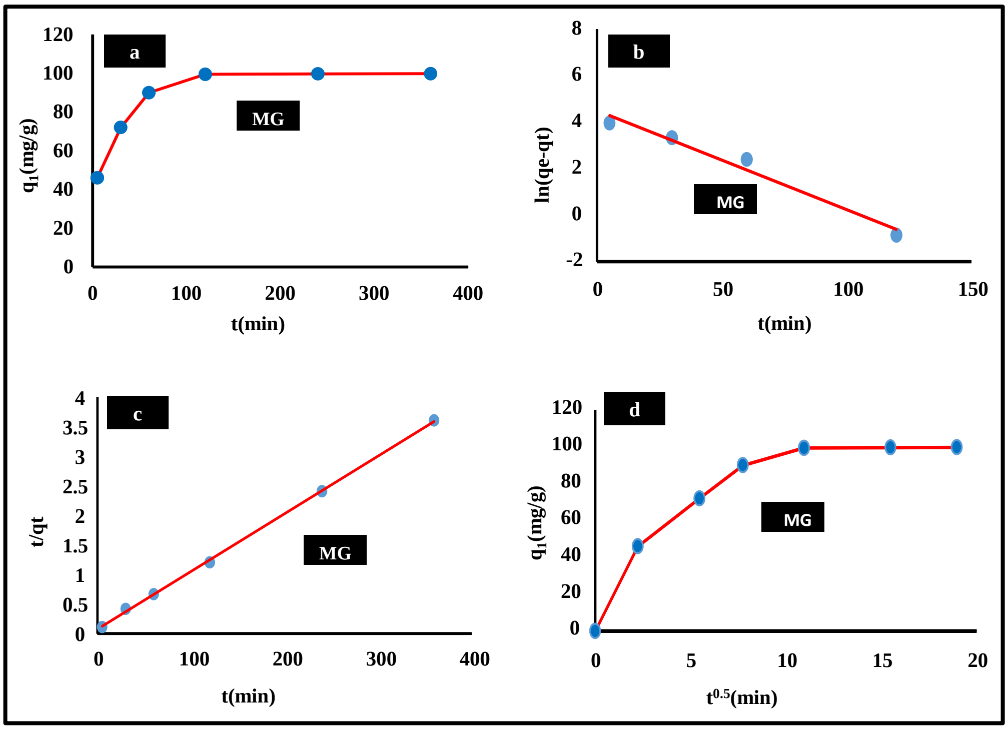

Figure S2. Effect of contact time (**a**), fitting of the kinetic data with pseudo-first order (**b**), pseudo-second order (**c**), and intra-particle diffusion model (**d**) on MG uptake.

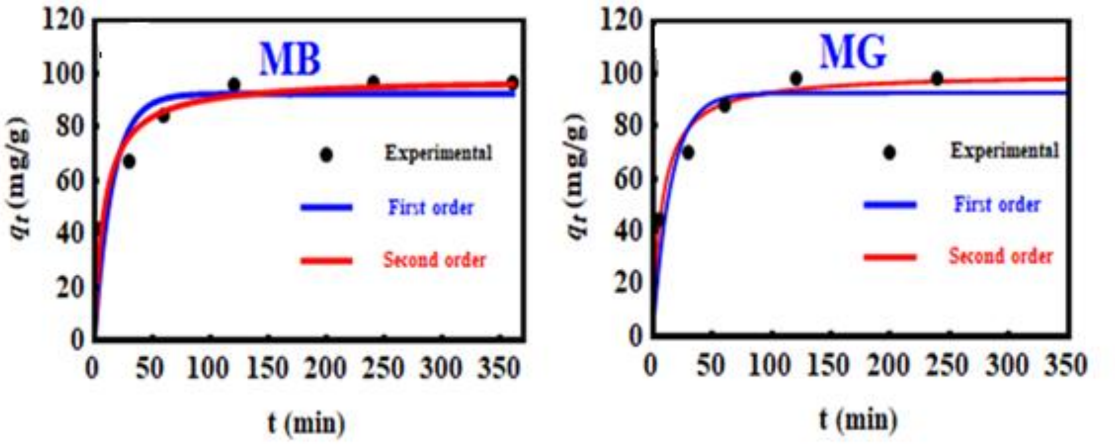

Figure S3. The non-linear plots of the pseudo-first order and the pseudo-second order kinetic models of MB and MG adsorption onto MNP/SP composite.

Table S2. Fitting the kinetic data with different kinetic models.

**Kinetic Model**		Parameters		R^2
**Pseudo-first order**		$q_{e\,(cal)}$ (mg/g)	k_1 (min^{-1})	
**MB**	Linear	108.88	0.0427	0.9358
	Non-linear	94.55	0.0646	0.9874
MG	Linear	111.41	0.0466	0.9424
	Non-linear	92.17	0.0857	0.9885
Pseudo-second order		$q_{e\,(cal)}$ (mg/g)	k_2 (g/mg min)	
MB	Linear	97.08	0.0012	0.9961
	Non-linear	98.44	0.0012	0.9963
MG	Linear	102.04	0.0013	0.9995
	Non-linear	100.14	0.0013	0.9969
Intra-particle diffusion		k_p (mg/g $min^{0.5}$)	C	
MB		4.576	30.065	0.732
MG		4.598	32.462	0.712

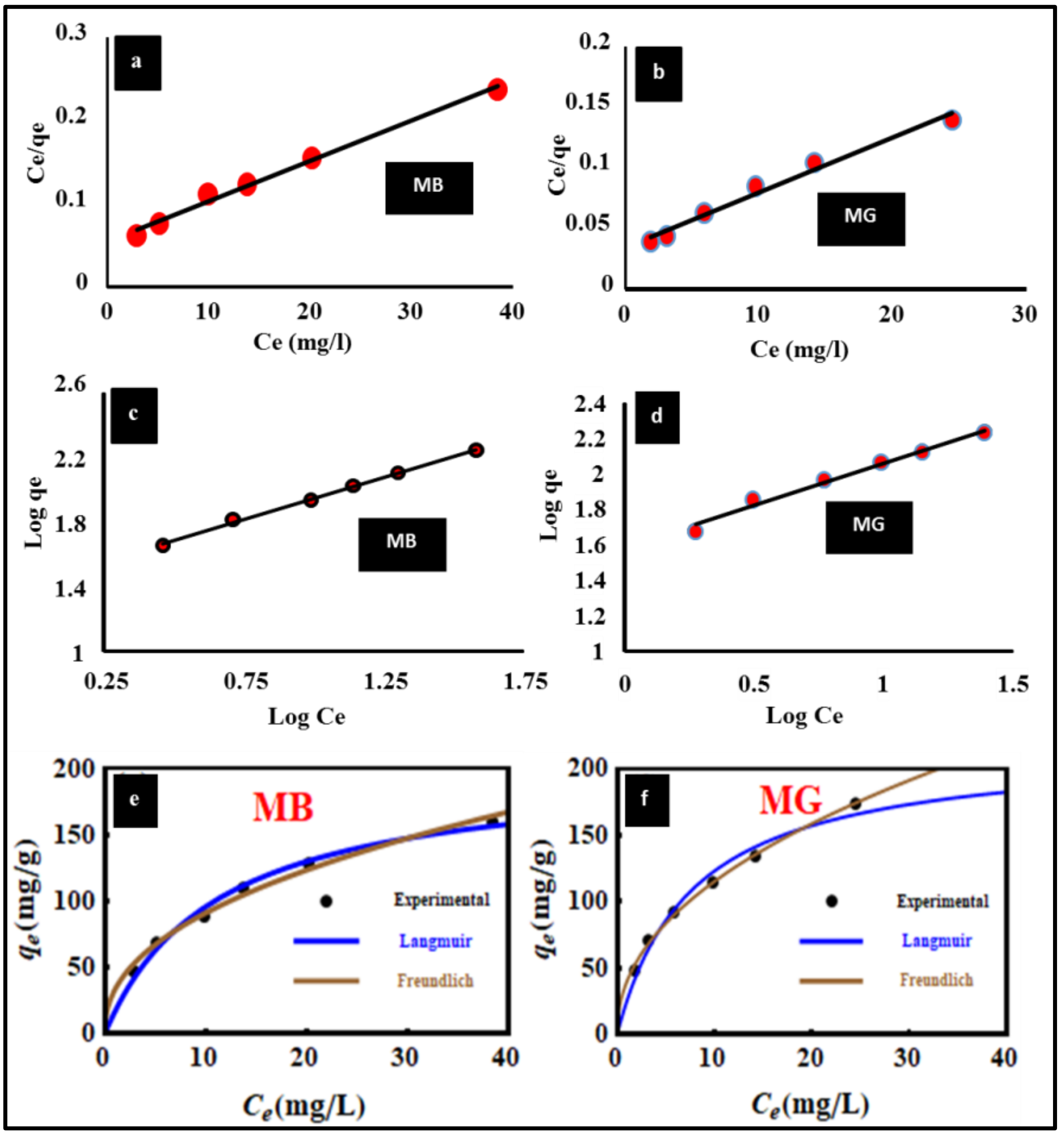

Figure S4. Linear Langmuir fitting (**a**, **b**), linear Freundlich fitting (**c**, **d**), and the non-linear fitting using Langmuir and Freundlich models (**e**, **f**).

Table S3. Parameters of isotherms models for MB and MG uptake by MNP/SP adsorbent.

**Isotherm Model Langmuir**		**Parameters** $q_{\max}$(mg/g)	K_L (L/mg)	R^2
**MB**	Linear	208.33	0.087	0.992
	Non-linear	201.25	0.091	0.998
**MG**	Linear	222.22	0.132	0.981
	Non-linea	217.64	0.130	0.997
**Freundlich**		K_F **(mg/g) (L/mg)$^{1/n}$**	$1/n$	
**MB**	Linear	27.18	0.519	0.997
	Non-linear	33.28	0.437	0.998
**MG**	Linear	38.68	0.211	0.986
	Non-linear	39.79	0.462	0.999

© 2020 by the authors. Submitted for possible open access publication under the terms and conditions of the Creative Commons Attribution (CC BY) license (http://creativecommons.org/licenses/by/4.0/).
